# Effects of Exogenous Glucocorticoid Infusion on Appetitic Center Development in Postnatal Dairy Bull Calves

**DOI:** 10.3390/ani13121980

**Published:** 2023-06-14

**Authors:** Keelee J. McCarty, Scott L. Pratt, Nathan M. Long

**Affiliations:** Animal And Veterinary Science Department, Clemson University, Clemson, SC 29631, USA

**Keywords:** exogenous glucocorticoid, leptin, neuronal development, appetite regulation, dairy calves

## Abstract

**Simple Summary:**

The profitability of production systems for livestock is directly impacted by body weight, which is influenced by an individual animal’s hypothalamic regulation of food intake. The objective of this study was to determine the effects of exogenous cortisol administration on circulating leptin concentrations, protein expression in various fat depots, and the hypothalamic expression of genes associated with appetite regulation in Holstein bull calves. Within 4 h of parturition, Holstein bull calves (9/treatment) were intravenously infused with either a low (3.5 µg/kg of body weight (BW)) or high (7.0 µg/kg of BW) dose of cortisol or a sham infusion control (with a similar volume of saline). At five days of age, blood, cerebrospinal fluid from the third ventricle of the brain, and adipose (omental, perirenal, and mesenteric) and hypothalamic tissue were collected for the analysis of proteins and genes associated with appetite regulation. Exogenous cortisol administered to perinatal dairy bull calves reduced leptin concentrations in serum and cerebrospinal fluid, decreased the protein expression of leptin in perirenal and omental adipose tissue, and altered gene expression in hypothalamic tissue. Further investigation is necessary to determine if glucocorticoid administration can be utilized as a tool to improve feed intake in cattle later on in life due to hypothalamic programming at birth.

**Abstract:**

The objective of this study was to determine the effects of exogenous glucocorticoid administration on leptin concentrations and brain development markers, such as protein and hypothalamic gene expression, in dairy bull calves. Within 4 h of parturition, Holstein bulls were intravenously infused with either a low cortisol dose (LC; *n* = 9, 3.5 µg/kg of body weight (BW)), high cortisol dose (HC; *n* = 9, 7.0 µg/kg BW), or control (CON; *n* = 9, saline) dose, with a 2nd infusion 24 h postpartum. Jugular blood was collected prior to infusion and daily until the calves were euthanized (day 5). Cerebrospinal fluid (CSF) from the third ventricle and adipose (omental, perirenal, and mesenteric) and hypothalamic tissue were collected. The blood and CSF samples were analyzed for leptin concentrations. The data were analyzed using SAS. Serum (*p* = 0.013) and CSF (*p* = 0.005) leptin concentrations in HC- and LC-treated calves were decreased compared with CON-treated calves. Leptin protein expression was decreased (*p* < 0.044) in perirenal and omental adipose tissue of LC-treated calves compared with CON-treated calves. Gene abundance of brain-derived neurotrophic factor and fibroblast growth factors 1 and 2 were decreased (*p* < 0.006) in HC- and LC-treated calves compared with CON-treated calves. In summary, cortisol administered to dairy bull calves reduced leptin concentrations, decreased leptin protein expression in perirenal and omental adipose tissue, and altered gene expression in hypothalamic tissue.

## 1. Introduction

The hypothalamic regulation of food intake and energy expenditure contributes to energy homeostasis by sensing circulating nutrients and hormones. Additionally, the hypothalamus receives and responds to afferent neuronal signals from peripheral tissues such as adipocytes, the pancreas, the gastrointestinal tract, and other brain regions [1]. The modulation of feeding behavior by both the central nervous system (CNS) and peripheral tissues is associated with the release and action of neuropeptides. For instance, neurons of the arcuate nucleus (ARC) release neuropeptides, such as orexigenic neuropeptide Y (NPY), to directly regulate energy expenditure through G-protein-coupled receptors [2,3] as well as interact with other portions of the brain. Due to their close proximity to the median eminence of the blood–brain barrier (BBB), the ARC interacts with the paraventricular nucleus (PVN) to regulate food intake. A suggested mechanism for this regulation is that ARC neurons sense leptin, an adipokine that acts via the obese gene (Ob), which has the capability to cross the BBB and bind to its receptors within the CNS [4]. The detection of elevated leptin levels down-regulates feeding behavior and simultaneously stimulates energy expenditure using neural and endocrine pathways [5].

Interestingly, the neuronal circuitry of the brain is still forming during early postnatal life in mammals, and it has been suggested that leptin promotes the maturation of the hypothalamic centers controlling food intake [6,7,8]. Fetal stress initiates parturition [9,10,11], and following birth, cattle exhibit elevated circulating leptin concentrations from 1 to 4 days of age [12], while sheep have a surge from days 5 to 9 [13]. Decreased leptin concentrations reported during early development have been associated with increased feed intake in mature ruminants [12,13]. Therefore, it is imperative to understand the mechanism behind feeding behavior as it directly impacts the profitability of cattle in production systems by influencing voluntary feed intake and body composition and altering subsequent feed costs. For example, offspring born to nutrient-restricted dams had decreased leptin and increased cortisol concentrations at birth [14] followed by increased feed consumption during a 10 wk ad libitum feeding trial [15].

Administration of a glucocorticoid has been suggested in place of a nutritional stimulus or insult during gestation. Glucocorticoids have been reported to decrease leptin concentrations and may serve as a management tool as a means to alter endocrine status and associated brain development. Previously, Long and Schafer [12] observed that cattle exhibit increased leptin from approximately 1 to 4 days of postnatal life concomitant with elevated cortisol at birth that decrease over the next 5 days of life. Mid-gestation administration of synthetic glucocorticoids, such as dexamethasone, increased cortisol and decreased leptin concentrations in grand offspring [16]. Additionally, ruminants with increased cortisol in the first 24 to 48 h of life had decreased serum leptin concentrations throughout the early postnatal period [12,13,17,18].

Glucocorticoids have been proven to alter leptin concentrations during gestation; however, this method may be too costly or labor-intensive for animal producers to incorporate as a management tool. As a replacement for administration throughout gestation, the use of an exogenous glucocorticoid infusion at birth in cattle has been suggested and proven to alter leptin concentrations from days 1 to 17 of age in early postnatal beef calves [19]. Glucocorticoid administration may serve as a model for hypothalamic programming in early postnatal ruminants through the repression of circulating leptin, thus stimulating feeding behavior, but the contributors to this mechanism of action remain unclear. The objective of this study was to determine the effects of exogenous cortisol administration on circulating leptin concentrations, protein expression in various fat depots, and the hypothalamic expression of genes associated with appetite regulation. Our hypothesis was that exogenous glucocorticoid administration would reduce circulating and cerebrospinal fluid leptin concentrations and that this would alter gene expression in the hypothalamus.

## 2. Materials and Methods

### 2.1. Animal Care and Treatments

All procedures were approved by Clemson University’s Institutional Animal Care and Use Committee (AUP #2017-05553).

At birth, bull calves (*n* = 27) born from multiparous Holstein Friesian cows (≤2.5 body condition score (−1/5 scale) and no problem calving; <2 dystocia score) were obtained from Clemson University Lemaster Dairy Center (Clemson, SC, USA). Within 4 h of parturition, calf body weight (BW) was determined via a hoof circumference measuring tape (Calfscale Company, Ames, IA, USA). Blood samples were collected using 10 mL tubes containing EDTA (Sarstedt, Newton, NC, USA) via jugular venipuncture prior to treatment infusion. Calves were randomly assigned into one of three treatment groups. Calves were then given an intravenous infusion over several seconds of either hydrocortisol sodium succinate (Solu-Cortef; Pfizer, New York, NY, USA) or sterile saline depending on the treatment group. The dosages of the cortisol infusions were based on calf BW, whereby calves received 3.5 μg/kg of BW of hydrocortisol sodium succinate (*n* = 9; low cortisol (LC)), 7.0 μg/kg of BW of hydrocortisol sodium succinate (*n* = 9; high cortisol (HC)), or a similar volume (4 to 11 mL) of sterile saline as for the LC-treated calves (*n* = 9; control (CON)). At 24 ± 4 h of age, a 2nd blood sample was collected, and each calf received a 2nd intravenous infusion of either LC at a dosage of 1.5 μg/kg of BW, HC at a dosage of 3.0 μg/kg of BW, or CON with a similar volume of sterile saline as for the LC dosage. The low cortisol dose was calculated to mimic the biological secretion of cortisol concentrations reported by LeMaster et al. [14] and the high cortisol dose was double the low dose. Pooled colostrum from the farm was provided immediately following blood collection and cortisol infusion following parturition, and then the calves were housed identically and fed a milk replacer (28% CP, 20% fat) 3 times daily with ad libitum access to water.

#### 2.1.1. Blood Collection

The remaining blood samples were collected via jugular venipuncture daily at 0600 h from days 2 to 5 of age (prior to the first meal of the day) and were immediately placed on ice. Serum samples were collected on day 5 prior to euthanasia and the collection of tissue samples. Serum samples were collected in 9 mL serum collection tubes (Sarstedt, Newton, NC, USA). The tubes were then incubated at room temperature for 1 h and then overnight at 4 °C. After overnight incubation, the serum samples were centrifuged at 1800× *g* for 20 min at 4 °C. Serum was decanted and stored long-term at −20 °C until analysis.

#### 2.1.2. Tissue Collection

At 100 ± 4 h (approximately 5 days of age), calves were euthanized utilizing a bolus jugular infused overdose of sodium pentobarbital (86 mg/ kg BW; Beuthanasia-D Special; Schering-Plough Animal Health, Union, NJ, USA). Immediately following euthanasia, the brains were extracted to collect hypothalamic and pituitary samples as well as extract cerebrospinal fluid (CSF) from the third ventricle of the brain for the quantification of leptin content and Western blotting. To determine protein expression, all adipose tissue samples were snap-frozen in liquid nitrogen, and the collection was performed via previously published procedures [20]. Briefly, adipose samples for perirenal (PR), omental (OM), and mesenteric (MES) depots were collected. OM adipose tissue was collected close to the dorsal rumen and the celiac artery, PR adipose tissue from around the left kidney close to the hilus, and MES adipose tissue between the cecum and the colon. All samples, following freezing, were stored long-term at −80 °C until analysis.

### 2.2. Hormone Analysis

Serum leptin concentrations were determined via radioimmunoassay in duplicate in multiple assays (Multispecies leptin RIA, Linco Research, St. Charles, MO, USA) within a single assay and with a sensitivity of 0.5 ng/mL using a previously validated procedure [12]. CSF concentrations were determined via radioimmunoassay in duplicate in multiple assays (Multispecies leptin RIA, Linco Research, St. Charles, MO, USA) within a single assay. Validation of the CSF analysis was performed to mimic the procedure performed by Long and Schafer [12]. Briefly, the volume of CSF was doubled, and 10 samples were analyzed to be corrected back to the linear range provided in the standard curve. A 99.1% recovery of mass was observed with a sensitivity of 0.12 ng/mL.

### 2.3. Western Blotting Analysis

Western blot analyses were conducted according to previously established methods [21]. Briefly, protein was extracted from ~200 mg of pulverized PR, OM, and MES adipose tissue depots using an ice-cold lysis buffer and a mechanical homogenizer (Tissue Tearor, Biospec Productions Inc., Bartlesville, OK, USA). Homogenates were sonicated and clarified via centrifugation, and the protein concentration was then determined using the Bradford protein assay (Bio-Rad Laboratories, Hercules, CA, USA). The supernatant was mixed with 2.5 × SDS sample loading buffer for a 20 µL total loading volume per well. Protein extractions were separated using 12% mini-PROTEAN TGX precast gels (Bio-Rad Laboratories, Hercules, CA, USA) followed by transfer to a 0.2 µm nitrocellulose membrane (Bio-Rad Laboratories, Hercules, CA, USA) for immunoblotting analyses. All blots were washed 3 times and incubated in a blocking solution of 5% bovine serum albumin (BSA) in tris-buffered saline (TBS) containing 0.1% Tween20 solution (TBST). The blots were incubated in primary antibodies (Ab) against leptin (Ob; ab16227; abcam, Cambridge, MA, USA) and glucocorticoid receptors (GR; ab3578; abcam, Cambridge, MA, USA) diluted in a blocking solution at a 1:1000 dilution. The blots were incubated overnight at 4 °C, washed with a blocking solution, followed by a final 1 h incubation at room temperature using a secondary Ab, donkey anti-rabbit IgG (A16035; Invitrogen, Carlsbad, CA, USA), diluted in a blocking solution at a 1:1000 dilution. The blots were washed with TBST for 5 min, and membrane stripping was performed via 2 washes with glycine (400 nM, pH 2.5) for 15 min each at room temperature, followed by 2 PBS washes and a TBST wash for 10 min each. Chemiluminescent detection was performed using the ChemiDoc MP Gel Imaging system (Bio-Rad Laboratories, Hercules, CA, USA). Each membrane (per adipose tissue depot) was first used for the detection of the target protein and then underwent glycine stripping and was re-probed for the housekeeping gene. Following membrane stripping, the blots were incubated in the primary antibody for glyceraldehyde 3 phosphate dehydrogenase (GAPDH; 14C10; Cell Signaling Technologies, Danvers, MA, USA) diluted in a mixture of 5% non-fat dry milk and a blocking solution at a 1:1000 dilution. The blots were incubated overnight at 4 °C, washed in a 5% non-fat dry milk and a blocking solution, followed by a final 1 h incubation at room temperature using the secondary Ab and immunodetection as previously mentioned. Protein was quantified via band density measurements of the Western blots. The densitometry data for leptin and GR protein expression were normalized to a GAPDH housekeeping protein density as follows. Background subtraction was applied to each GAPDH, Ob, and GR sample density. Blot normalization factors were calculated for each blot (the highest GAPDH expression value on the blot/the GAPDH signal of each individual sample). The adjusted density of each sample for Ob and GR signals was then divided by the blot normalization factor to obtain the relative protein expression.

### 2.4. Real-Time RT-PCR

Total RNA was extracted from liquid-nitrogen-powdered hypothalamic tissue using Tri^®^Reagent (Invitrogen, Carlsbad, CA, USA), and cleanup was performed with the E.Z.N.A RNA clean-up kit (Omega-Biotek; Norcross, GA, USA). Extracted total RNA purity was assessed using a NanoDrop One spectrophotometer (Thermo Fisher Scientific, Wilmington, DE, USA) as well as the visible quantification of ribosomal RNA bands. Samples exhibiting 260 nm/280 nm ratios between 1.9 and 2.1 were deemed acceptable for downstream procedures. cDNA was synthesized using SuperScriptTM III first-strand synthesis for an RT-PCR kit (Invitorgen, Carlsbad, CA, USA). Real-time RT-PCR was conducted using the CFX96 Touch Real-Time PCR detection system (Bio-Rad Laboratories, Hercules, CA, USA) in which beta-actin (ACTB) and GAPDH served as housekeeping genes. In hypothalamic samples, the genes of interest associated with neuronal growth factors and dendritic growth were leptin (Ob), leptin receptor (ObR), glucocorticoid receptor (GR), brain-derived neurotrophic factor (BDNF), fibroblast growth factor-1 (FGF1), and fibroblast growth factor-2 (FGF2). All primer pairs and generated products were verified, and the accession numbers, primer sequences, and product sizes for all primer combinations are given in Table 1. For qRT-PCR, pooled RNA (1 µg) was reverse-transcribed as stated above. A standard curve based on the original mass of the RNA in the RT reaction was generated (50, 25, 12.5, 6.35, and 3.125 ng per reaction), run in duplicate, and subjected to qRT-PCR using the SsoAdvanced Universal SYBR Green SuperMix kit (Bio-Rad Laboratories, Hercules, CA, USA) using a CFX96 Touch Real-Time PCR detection system (Bio-Rad Laboratories, Hercules, CA, USA). Primer efficiency was calculated via regression analysis. The thermal cycling conditions for qRT-PCR were as follows: polymerase activation and DNA denaturation at 95 °C for 30 s 40 PCR cycles at 95 °C for 10 s, and an annealing temperature specific to the primer for 30 s, as reported in Table 1. Melting curves were generated at the end of amplification, at 50–95 °C in 0.5 °C increments, to verify the presence of a single product. Data normalization was performed using the averages of the housekeeping genes ACTB and GAPDH. The cycle thresholds (CTs) for the housekeeping genes and all the target genes were used for normalization via repeated pair-wise correlation and regression analysis [21,22]. Normalized CT values (ΔCT = CT,gene − CT,Housekeeping) were calculated for each sample, and fold changes in gene expression were calculated using the X−ΔΔCT method, where X is the primer efficiency for each gene of interest via previously established methods [23].

### 2.5. Statistical Analysis

Calf birth weights were analyzed via ANOVA (SAS software version 9.4, SAS Institute, Cary, NC, USA). The model statement included treatment. The blood analyses of serum leptin concentrations were analyzed via MIXED SAS procedures. The model statement included the day, treatment, and their respective interactions. Analyses of CSF leptin concentrations were analyzed via GLM SAS procedures. The model statement included treatment. Individual calves served as the experimental unit for body weight and hormone analysis. The area under the curve (AUC) was determined using GraphPad Prism for serum leptin concentrations from days 0 to 5 of age. Pearson correlation coefficients were derived using the CORR SAS procedure to analyze the relationships between serum leptin concentrations from days 0 to 5 of age and CSF leptin concentrations. Analyses of the protein expressions of mesenteric, omental, and perirenal AT were analyzed via GLM SAS procedures. The model statement included treatment. For qRT-PCR, expression differences were determined by subtracting the CTs of target genes from the CTs of the averaged housekeeping genes (ACTB and GAPDH), and the differences were analyzed via GLM SAS procedures. The model statement included treatment. Statistical significance was declared at *p* ≤ 0.05.

## 3. Results

### 3.1. Calf Morphometric Results

**Figure 1:**
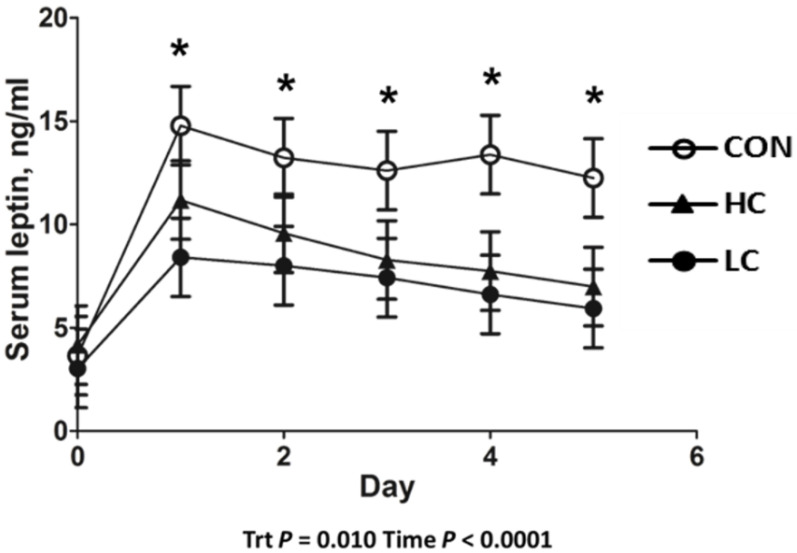
Leptin radioimmunoassay results of serum samples collected from 0 to 5 days of age from calves intravenously infused with either a low cortisol dose (LC; *n* = 9, 3.5 µg/kg of BW), high cortisol dose (HC; *n* = 9, 7.0 µg/kg of BW), or a sham infusion control (CON; *n* = 9; similar volume of saline). (*) indicates a treatment x time difference (*p* < 0.05); Trt = treatment effects.

**Figure 2:**
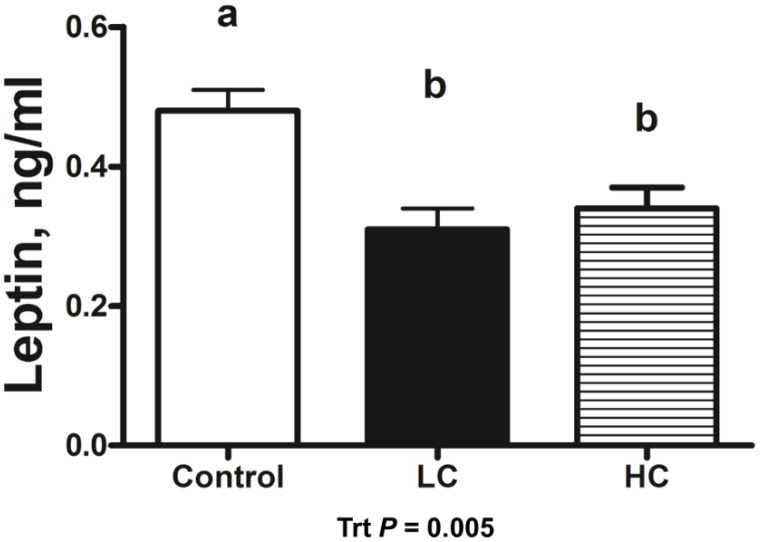
Leptin radioimmunoassay results of cerebrospinal fluid (CSF) samples collected at 5 days of age from calves intravenously infused with either a low cortisol dose (LC; *n* = 9, 3.5 µg/kg of BW), high cortisol dose (HC; *n* = 9, 7.0 µg/kg of BW), or a sham infusion control (CON; *n* = 9; similar volume of saline). (a,b) indicates means differ (*p* < 0.05); Trt = treatment effects.

Calf birth weight did not differ (*p* = 0.750) between CON- (39.2 ± 1.5 kg), LC- (40.2 ± 1.5 kg), or HC- (38.5 ± 1.5 kg) treated calves. Serum leptin concentrations in HC- and LC-treated calves were decreased (*p* = 0.013) compared with CON-treated calves. Day 0 serum leptin was decreased (*p* < 0.001) compared with all other days of age (Figure 1). Leptin concentrations in CSF decreased (*p* = 0.005) in HC- and LC-treated calves compared with CON-treated calves at day 5 of age (Figure 2). A moderate positive correlation (r = 0.559; *p* = 0.002) was observed between CSF and day-5 serum leptin concentrations. A strong positive correlation (r= 0.697; *p* < 0.001) was observed between CSF and the AUC of serum leptin concentrations from 1 to 5 days of age.

### 3.2. Protein Expression Results

**Figure 3:**
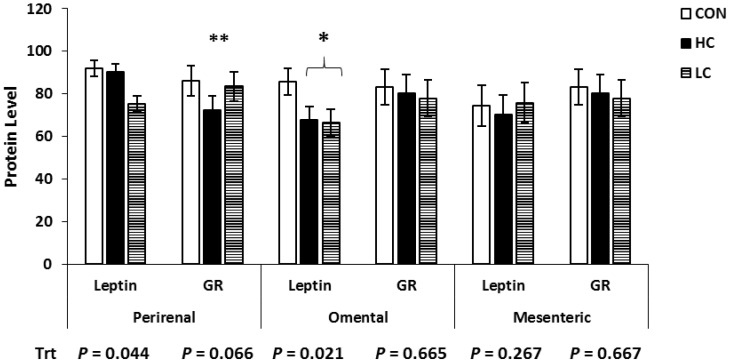
Western blot analysis of adipose tissue samples collected at 5 days of age from calves intravenously infused with either a low cortisol dose (LC; *n* = 9, 3.5 µg/kg of BW), high cortisol dose (HC; *n* = 9, 7.0 µg/kg of BW), or a sham infusion control (CON; *n* = 9; similar volume of saline). Protein was quantified via band densitometry of the Western blots, and densities were normalized to glyceraldehyde 3 phosphate dehydrogenase (GAPDH) content within each sample. * Means ± SEM differ (*p* < 0.05); ** means ± SEM tend to differ (*p* < 0.10); Trt = treatment effects.

The Western blot analysis for Ob and GR in various adipose tissue depots is shown in Figure 3. Protein expression of Ob was decreased (*p* < 0.044) in the PR and OM adipose tissue of LC-treated calves compared with CON-treated calves, while MES did not differ between treatments (*p* = 0.267). Protein expression of GR tended (*p* = 0.067) to be decreased in the PR tissue of HC-treated calves compared with CON-treated calves and did not differ (*p* > 0.665) between treatments in other adipose tissue depots.

### 3.3. Gene Expression Results

The genes of interest associated with neuronal growth factors and dendritic growth in hypothalamic samples were Ob, ObR, GR, BDNF, FGF1, and FGF2. The mean relative normalized expression levels of mRNA for the genes of interest in hypothalamus samples collected at 5 days of age in calves are reported in Table 2. The hypothalamic expressions of Ob, ObR, and GR were not different between treatments (*p* > 0.224). Expression of BDNF was decreased (*p* = 0.003) in LC samples compared with CON samples, FGF1 was decreased (*p* = 0.0004) in HC and LC samples compared with CON samples, and FGF2 was decreased (*p* = 0.006) in LC samples compared with CON hypothalamus samples.

## 4. Discussion

### 4.1. Calf Morphometric Measurements and Hormone Analysis

In the current experiment, animals were randomized per treatment group and had similar BWs across treatments. Lewis et al. [19] performed the administration of hydrocortisol sodium succinate at 3.5 μg/kg of BW within 4 h of birth in Angus calves from dams of a similar BCS. The birth weights were not different between treatments (average of 38.3 ± 1.4 kg), cortisol-treated calves had decreased leptin concentrations from 1 to 13 days of age, and there were no differences in BW from 60 to 150 days of age [19]. Limited research has been performed on exogenous glucocorticoid administration at birth and postnatal leptin secretions in beef cattle. Previously, administration of a synthetic glucocorticoid (dexamethasone) in perinatal dairy calves at 15 μg/kg of BW did not alter leptin concentrations from 1 to 4 days of age [24]. However, in the study by Blum et al. [24], calves were supplemented with dexamethasone in their colostrum, and it may not have elicited an effect based on the amount that traversed the gastrointestinal tract to general circulation. In lambs, infusion of exogenous cortisol at birth and 24 h of life resulted in a decreased leptin concentration in a dose-dependent manner [25].

The transfer of leptin from the peripheral blood to CSF occurs within the choroid plexus (CP) of the brain (within the third ventricle). The ARC lies adjacent to the median eminence, which contains hypothalamic glia that line the third ventricle, and serves as the CSF barrier. Peripheral blood that has entered through the pituitary portal system is filtered through the blood–CSF barrier. In the current study, both peripheral blood and CSF concentrations of leptin were decreased in the glucocorticoid-treated groups, irrespective of dosage. Furthermore, a strong positive correlation was observed between CSF leptin concentrations and the AUC of circulating leptin concentrations from 1 to 5 days of age. Adam and Findlay [26] utilized an obese sheep model to determine if leptin resistance was due to decreased blood–brain leptin transfer or hypothalamic insensitivity. Briefly, sheep were sorted by leanness (obese versus lean) as well as being administered leptin (0.5 mg of leptin/kg BW) via intracerebroventricular (ICV) cannulae to detect leptin concentrations in blood and CSF. Obese animals had no difference in the leptin sensitivity of the brain but did have impaired leptin transport across the BBB [26]. Leptin resistance may be induced by a lack of downstream signaling of leptin binding to neuronal receptors as a means of inducing obesity (associated with impaired leptin signaling) or the decreased efficiency of BBB leptin transporters [27]. Disrupted signal transduction pathways may be due to uncoupled leptin from ObR due to increased leptin concentrations, as observed in obese patients [28]. Ishida-Takahasi et al. [29] performed a similar experiment in rodents in which animals received a dexamethasone ICV infusion prior to a leptin ICV infusion. Observations in the study included the antagonistic action of dexamethasone on leptin via impaired leptin-induced signaling pathways JAK/STAT and MAPK signaling cascades [29]. Overall, reduced leptin concentrations in circulation lead to reduced amounts of leptin traversing the BBB (and reduced CSF concentrations). This means that leptin concentrations in the brain would be depressed, thus diminishing the amount of influence it would have on brain development during early postnatal life as well as potentially impairing signaling pathways. The current experiment evaluated the protein and mRNA expression of leptin and glucocorticoid receptors but did not evaluate the presence or activity of leptin transporters. Thus, more experimentation is required to understand the blood–CSF transfer and the effects on downstream intracellular leptin signaling pathways in hypothalamic development in cattle.

### 4.2. Protein Expression

Both Ob and ObR have been previously detected in adipose tissue depots and hypothalamus samples in dairy cattle [30], which supports the idea that leptin may have multiple physiological functions in cattle. Conversely, Ji et al. [28] did not observe Ob expression in the brains of beef cattle. Bovine adipose tissue treated with dexamethasone (100 nM) in vitro stimulated Ob [31,32], while ad libitum feeding in late gestation reduced Ob expression in adipose tissue in sheep [33]. Under control conditions in which energy requirements are maintained in dairy cattle, Ob and ObR expressions have similar expressions in various adipose tissue depots [30]. Based on the responses of all of the adipose tissue depots to glucocorticoid administration in the current study, PR adipose tissue may play a role in the leptin signaling cascade as Ob was decreased and GR tended to be decreased. This may be due to the fact that PR adipose tissue was the largest depot present at the time of collection in the early postnatal calves. While OM adipose tissue is functional during early postnatal life, due to its role in thermoregulation, it may not be sensitive or mature enough to respond to glucocorticoids. Conversely, while subcutaneous tissue is also associated with thermoregulatory action, it is present in limited quantities at the time of birth and may not support leptin production. Following administration, the exogenous glucocorticoid binds to PR adipose tissue receptors, decreasing leptin production, which ultimately reduces the leptin in general circulation. Overall, this supports the hypothesis that PR adipose tissue is the primary source of leptin production in postnatal ruminants that contributes to the increased leptin circulation during the early period of postnatal life previously reported by Long and Schafer [12].

### 4.3. Gene Expression

As previously mentioned, the connection and development of neuronal circuitry in the brain are still forming during the first few days of postnatal life and play a role in appetite regulation. The greatest expression of ObR has previously been observed in the CNSs of monogastric species [34]. However, the current study utilized a single form of the leptin receptor (ObR) rather than separating it into the short form (Ob-Ra) or long form (Ob-Rb) of the receptor. This is due to the fact that only the long form has been observed to be fully functional and contribute to the effectiveness of leptin signaling [35], whereas the short form of the receptor contributes to leptin transport [36] and catecholamine synthesis [37]. In the current study, the expression of Ob did not differ due to treatment, suggesting that local hypothalamic expression was not altered. This may be due to the fact Ob and ObR are already highly expressed in the brain [35] and the body may be trying to maintain homeostasis as leptin is associated with metabolic regulation and energy expenditure. Similar profiles are observed in human, rat, and mouse obesity models in which decreased leptin expression is a result of increased adipose Ob mRNA and serum leptin levels [38,39,40,41,42].

Similar to Ob and ObR, GR expression in the hypothalamus did not differ in the current study. In the literature, most administration models for glucocorticoids in livestock species are performed during gestation. As glucocorticoids are primarily associated with the maturation of specific fetal tissues during late gestation, in the findings of Liggins [43], dexamethasone administration improved the mortality and morbidity of premature sheep offspring relative to lung maturation. Ovine and rodent models of maternal synthetic glucocorticoid administration (dexamethasone and betamethasone, respectively) during gestation altered GR mRNA expression throughout the adulthood of subsequent offspring [44,45]. Models of maternal undernutrition in sheep reported altered pituitary and adrenal GR, hypothalamic corticotropic-releasing hormone, and adrenocorticotropic hormone mRNA abundance [1,46]. However, unaltered glucocorticoid expression in the brain may be beneficial for the health of the animal as permanently altered GR expression may impair the hypothalamic–pituitary–adrenal axis (HPAA) and associated stress response. The HPAA is susceptible to stress induced by the maternal environment, which impairs responsiveness in maturity [47,48].

In the current study, the hypothalamic gene expressions of BDNF, FGF1, and FGF2 were decreased in cortisol-treated calves compared with control-treated calves. Brain-derived neurotrophic factor (BDNF), which is encoded by the BDNF gene, is a protein that promotes differentiation [49], maturation [50], and the maintenance of neurons [51], and stimulates neurogenesis during development [52,53]. Exogenous supplementation with BDNF in tissue cultures increased dendritic length as well as modulating specific branching and growth patterns, but only when neurons were active enough to respond to the action of BDNF [54,55,56,57]. Fibroblast growth factors (FGFs) stimulate and differentiate various endothelial cells, such as mesoderm- and neuroectoderm-derived cells, as well as promoting cell migration [58]. Both acidic (FGF1/FGFa) [58] and basic (FGF2/FGFb) [59] forms of FGFs play a role in endothelial cell development. Acidic FGFs have only been detected in the brain and retina [60,61,62,63], whereas basic FGFs have been found in the central nervous system and peripheral tissues.

Both BDNF and FGFs are associated with the development of neuronal circuitry that impacts appetite regulation. For example, the formation of ARC projections [5] is concomitant with elevated circulating leptin concentrations [6] in mice. The centers of the brain, such as the ARC, contain neurons that traverse the blood–brain barrier and come into contact with the bloodstream. The ARC responds to leptin [5] and expressed NPY, agouti-related peptide (Agrp), and anorexigenic proopiomelanocortin (POMC) as a means to regulate energy expenditure [2,3]. Maternal nutrient restriction in rats inhibits the formation of ARC projections [64], which is potentially due to a repressed leptin surge. Conversely, the leptin surge is amplified and prolonged in the offspring of obese rats [65], while a premature surge can increase the density of the ARC in PVN projections [66].

It has previously been speculated that glucocorticoids interact with BDNF to affect central nervous system function [67]. Rodents that underwent chronic restraint tests did not show altered BDNF or BDNF receptor (BDNFR) expression; however, BDNF-induced glutamate release was decreased [68]. After reduced interaction with BDNFR, tropomyosin receptor kinase B (TrkB) was observed following glucocorticoid exposure to cortical neurons in rodents [69]. Numakawa et al. [70] determined that GR’s association with TrkB plays a role in BDNF-stimulated signaling. Interestingly, Longenecker et al. [71] reported that the growth of primary bovine endothelial cells was not influenced by glucocorticoids in the absence of FGFs. The growth of bovine smooth muscle cells was recovered with FGFs when added to the culture to prevent inhibition by glucocorticoids [71]. Additionally, the activity of FGFs on DNA synthesis was inhibited in adrenal cell lines when treated with glucocorticoids [58]. As the literature is lacking, more experimentation is required to elucidate the mechanism of glucocorticoids on neuronal growth factors in the ruminant brain and their effects on early postnatal growth and development, as well as if altered protein expressions of the previously mentioned genes of interest reflect the changes in mRNA abundance observed across treatments in the current study.

## 5. Conclusions

Exogenous cortisol administered to perinatal dairy bull calves reduced leptin concentrations in serum and CSF, decreased the protein expression of leptin in perirenal and omental adipose tissue, and altered gene expression in hypothalamic tissue. The administration of glucocorticoids decreased circulating leptin concentrations, which then reduced the expressions of genes associated with the neuronal development of appetite control centers in the hypothalamus. This information leads to the speculation that reduced appetite center development in the hypothalamus leads to increased feed intake later in life due to the diminished neuronal regulation of appetite and satiety. The authors believe this to be the first study on exogenous glucocorticoid administration in cattle to be associated with altered brain development markers such as protein expression and mRNA abundance of hypothalamic gene expression related to appetite regulation during early postnatal life. The authors acknowledge the pleiotropic effect of glucocorticoids during this period of development. Because of this, further investigation is necessary to determine if glucocorticoid administration can be utilized as a tool to improve feed intake in cattle later on in life due to hypothalamic programming at birth.

## Figures and Tables

**Table 1:** Primers used for RT-PCR of genes of interest associated with dendritic and synaptic development in hypothalamus samples collected at 5 days of age from calves intravenously infused with either a low cortisol dose (LC; *n* = 9, 3.5 µg/kg of BW), high cortisol dose (HC; *n* = 9, 7.0 µg/kg of BW), or a sham infusion control (CON; *n* = 9, with a similar volume of saline).

Gene	Primer ^1^	Sequence (5′–3′)	Length (bp)	Annealing Temp. (°C)
Ob ^2^	F	TTCCCTCTACTCCACCGAGG	123	60
Ob	R	GGACTTTGGGAAGAGAGGCC		
ObR ^3^	F	CTGCTCCCCCAGAAAGACAG		65
ObR	R	GCTGAGCTGACATTGGAGGT		
GR ^4^	F	CAACTCACACCTACGCTGGT	163	60
GR	R	TTGCCTTTGCCCATTTCACG		
BDNF ^5^	F	TACCTGGATGCCGCAAACAT	134	60
BDNF	R	CGACATGTCCACTGCAGTCT		
FGF1 ^6^	F	AGGGATTCCAATGGCAAGGG	166	60
FGF1	R	TCCTGCTGCTGAATGACCAG		
FGF2 ^7^	F	CACGACTGAGCGACTTCACT	103	55
FGF2	R	GACCCCATAGACAGCAGCTC		
ACTB ^8^	F	CTCTTCCAGCCTTCCTTCCT	178	55
ACTB	R	GGGCAGTGATCTCTTTCTGC		
GAPDH ^9^	F	GGGGTCATCATCTCTGCACCT	176	55
GAPDH	R	GGTCATAAGTCCCTCCACGA		

^1^ F, forward; R, reverse; ^2^ leptin (Ob); ^3^ leptin receptor (ObR); ^4^ glucocorticoid receptor (GR); ^5^ brain-derived neurotrophic factor (BDNF); ^6^ fibroblast growth factor-1 (FGF1); ^7^ fibroblast growth factor-2 (FGF2); ^8^ beta-actin (ACTB); ^9^ glyceraldehyde 3 phosphate dehydrogenase (GAPDH).

**Table 2:** Mean relative normalized expression levels of mRNA for the genes of interest in hypothalamus samples collected at 5 days of age from calves intravenously infused with either a low cortisol dose (LC; *n* = 9; 3.5 µg/kg of BW), high cortisol dose (HC; *n* = 9; 7.0 µg/kg of BW), or a sham infusion control (CON; *n* = 9; similar volume of saline).

	Treatment			*p*-Value	
	CON	LC	HC	SE	Trt
*n*	9	9	9	-	-
Ob ^1^	11.99	9.85	5.32	3.18	0.333
ObR ^2^	1.43	2.12	1.58	0.47	0.188
GR ^3^	1.74	2.89	1.51	0.31	0.224
BDNF ^4^	1.67	3.50	1.43	0.41	0.003
FGF1 ^5^	0.87	4.79	2.34	0.73	0.0004
FGF2 ^6^	4.48	12.49	2.50	1.91	0.006

Data are given as mean ΔCT normalized to housekeeping gene expression. Higher values indicate lower gene expression. ^1^ Leptin (Ob); ^2^ leptin receptor (ObR); ^3^ glucocorticoid receptor (GR); ^4^ brain-derived neurotrophic factor (BDNF); ^5^ fibroblast growth factor-1 (FGF1); ^6^ fibroblast growth factor-2 (FGF2).

## Data Availability

The data presented in this study are available on request from the corresponding author.

## References

[1] McMillen I.C., Robinson J.S. (2005). Developmental origins of the metabolic syndrome: Prediction, plasticity, and programming. Phys. Rev..

[2] Friedman J.M., Halaas J.L. (1998). Leptin and the regulation of body weight in mammals. Nature.

[3] Schwartz M.W., Woods S.C., Porte D., Seeley R.J., Baskin D.G. (2000). Central nervous system control of food intake. Nature.

[4] Faouzi M., Leshan R., Björnholm M., Hennessey T., Jones J., Münzberg H. (2007). Differential accessibility of circulating leptin to individual hypothalamic sites. Endocrinology.

[5] Martin-Gronert M.S., Ozanne S.E. (2005). Programming of appetite and type 2 diabetes. Early Hum. Dev..

[6] Yura S., Itoh H., Sagawa N., Yamamoto H., Masuzaki H., Nakao K., Kawamura M., Takemura M., Kakui K., Ogawa Y. (2005). Role of premature leptin surge in obesity resulting from intrauterine undernutrition. Cell Metab..

[7] Bouret S.G., Simerly R.B. (2006). Developmental programming of hypothalamic feeding circuits. Clin. Genet..

[8] Vickers M.H., Gluckman P.D., Coveny A.H., Hofman P.L., Cutfield W.S., Gertler A., Breier B.H., Harris M. (2008). The effect of neonatal leptin treatment on postnatal weight gain in male rats is dependent on maternal nutritional status during pregnancy. Endocrinology.

[9] Bouillon-Minois J.B., Trousselard M., Thivel D., Benson A.C., Schmidt J., Moustafa F., Bouvier D., Dutheil F. (2021). Leptin as a biomarker of stress: A systematic review and meta-analysis. Nutrients.

[10] Abdelnour S.A., Abd El-Hack M.E., Khafaga A.F., Arif M., Taha A.E., Noreldin A.E. (2019). Stress biomarkers and proteomics alteration to thermal stress in ruminants: A review. J. Therm. Biol..

[11] Martínez-Burnes J., Muns R., Barrios-García H., Villanueva-García D., Domínguez-Oliva A., Mota-Rojas D. (2021). Parturition in mammals: Animal models, pain and distress. Animals.

[12] Long N.M., Schafer D.W. (2013). Sex effects on plasma leptin concentrations in newborn and postnatal beef calves. Prof. Anim. Sci..

[13] Long N.M., Ford S.P., Nathanielsz P.W. (2011). Maternal obesity eliminates the neonatal lamb plasma leptin peak. J. Physiol..

[14] LeMaster C.T., Taylor R.K., Ricks R.E., Long N.M. (2017). The effects of late gestation maternal nutrient restriction with or without protein supplementation on endocrine regulation of newborn and postnatal beef calves. Theriogenology.

[15] Tipton J.E., Ricks R.E., LeMaster C.T., Long N.M. (2018). The effects of late gestation nutrient restriction of dams on heifer offspring intake, metabolites and hormones during an *ad libitum* feeding trial. J. Anim. Physiol. Anim. Nutr..

[16] Long N.M., Smith D.T., Ford S.P., Nathanielsz P.W. (2013). Elevated glucocorticoids during ovine pregnancy increase appetite and produce glucose dysregulation and adiposity in their granddaughters in response to *ad libitum* feeding at 1 year of age. Am. J. Obstet. Gynecol..

[17] Long N.M., George L.A., Uthlaut A.B., Smith D.T., Nijland M.J., Nathanielsz P.W., Ford S.P. (2010). Maternal obesity and increased nutrient intake before and during gestation in the ewe results in altered growth, adiposity, and glucose tolerance in adult offspring. JAS.

[18] Shasa D.R., Odhiambo J.F., Long N.M., Tuersunjiang N., Nathanielsz P.W., Ford S.P. (2015). Multigenerational impact of maternal overnutrition/obesity in the sheep on the neonatal leptin surge in granddaughters. Int. J. Obes..

[19] Lewis L.K., Ricks R.E., Long N.M. (2019). Manipulation of neonatal leptin profile via exogenous glucocorticoids in beef calves. Animal.

[20] Long N.M., Rule D.C., Tuersunjiang N., Nathanielsz P.W., Ford S.P. (2015). Maternal obesity in sheep increases fatty acid synthesis, upregulates nutrient transporters, and increases adiposity in adult male offspring after a feeding challenge. PLoS ONE.

[21] Long N.M., Rule D.C., Zhu M.J., Nathanielsz P.W., Ford S.P. (2012). Maternal obesity upregulates fatty acid and glucose transporters and increases expression of enzymes mediating fatty acid biosynthesis in fetal adipose tissue depots. JAS.

[22] Pfaffl M.W., Tichopad A., Prgomet C., Neuvians T.P. (2004). Determinutesation of stable housekeeping genes, differentially regulated target genes and sample integrity: BestKeeper—Excel-based tool using pair-wise correlations. Biotechnol. Lett..

[23] Pfaffl M.W., Horgan G.W., Dempfle L. (2002). Relative expression software tool (REST©) for group-wise comparison and statistical analysis of relative expression results in real-time PCR. Nucleic Acids Res..

[24] Blum J.W., Zbinden Y., Hammon H.M., Chilliard Y. (2005). Plasma leptin status in young calves: Effects of pre-term birth, age, glucocorticoid status, suckling, and feeding with an automatic feeder or bucket. DAE.

[25] Ford S.P., Odhiambo J.F., Walton M.A., Nathanielsz M.W. (2015). Elevating Blood Cortisol (CORT) Concentrations at Birth in Lambs Eliminates the Early Postnatal Leptin Surge. Reprod. Sci..

[26] Adam C.L., Findlay P.A. (2010). Decreased blood–brain leptin transfer in an ovine model of obesity and weight loss: Resolving the cause of leptin resistance. Int. J. Obes..

[27] Szczesna M., Zieba D.A. (2015). Phenomenon of leptin resistance in seasonal animals: The failure of leptin action in the brain. Domest. Anim. Endocrinol..

[28] Ahima R.S., Flier J.S. (2000). Adipose tissue as an endocrine organ. Trends Endo Met..

[29] Ishida-Takahashi R., Uotani S., Abe T., Degawa-Yamauchi M., Fukushima T., Fujita N., Sakamaki H., Yamasaki H., Yamaguchi Y., Eguchi K. (2004). Rapid inhibition of leptin signaling by glucocorticoids in vitro and in vivo. J. Biol. Chem..

[30] Chelikani P.K., Glimm D.R., Kennelly J.J. (2003). Tissue distribution of leptin and leptin receptor mRNA in the bovine. J. Dairy Sci..

[31] Ji S., Willis G.M., Scott R.R., Spurlock M.E. (1998). Partial cloning and expression of the bovine leptin gene. Anim. Biotechnol..

[32] Houseknecht K.L., Portocarrero C.P., Ji S., Lemenager R., Spurlock M.E. (2000). Growth hormone regulates leptin gene expression in bovine adipose tissue: Correlation with adipose IGF-1 expression. J. Endocrinol..

[33] Bispham J., Gopalakrishnan G.S., Dandrea J., Wilson V., Budge H., Keisler D.H., Broughton Pipkin F., Stephenson T., Symonds M.E. (2003). Maternal endocrine adaptation throughout pregnancy to nutritional manipulation: Consequences for maternal plasma leptin and cortisol and the programming of fetal adipose tissue development. Endocrinology.

[34] Frühbeck G. (2001). A heliocentric view of leptin. Proc. Nutr. Soc..

[35] Tartaglia L.A. (1997). The leptin receptor. J. Biol. Chem..

[36] Hileman S.M., Pierroz D.D., Masuzaki H., Bjørbæk C., El-Haschimi K., Banks W.A., Flier J.S. (2002). Characterizaton of short isoforms of the leptin receptor in rat cerebral microvessels and of brain uptake of leptin in mouse models of obesity. Endocrinology.

[37] Yanagihara N., Utsunomiya K., Cheah T.B., Hirano H., Kajiwara K., Hara K., Nakamura E.I., Toyohira Y., Uezono Y., Ueno S. (2000). Characterization and functional role of leptin receptor in bovine adrenal medullary cells. Biochem. Pharmacol..

[38] Maffei Á., Halaas J., Ravussin E., Pratley R.E., Lee G.H., Zhang Y., Fei H., Kim S., Lallone R., Ranganathan S. (1995). Leptin levels in human and rodent: Measurement of plasma leptin and ob RNA in obese and weight-reduced subjects. Nat. Med..

[39] Frederich R.C., Hamann A., Anderson S., Lollman B. (2005). Leptin levels reflect body lipid content in mice: Evidence for diet-induced resistance to leptin action. Nat. Med..

[40] Considine R.V., Sinha M.K., Heiman M.L., Kriauciunas A., Stephens T.W., Nyce M.R., Ohannesian J.P., Marco C.C., McKee L.J., Bauer T.L. (1996). Serum immunoreactive-leptin concentrations in normal-weight and obese humans. N. Engl. J. Med..

[41] Hamilton B.S., Paglia D., Kwan A.Y.M., Dietel M. (1995). Increased obese mRNA expression in omental fat cells from massively obese humans. Nat. Med..

[42] Lonnqvist F., Arner P., Nordfors L., Schalling W. (1995). Overexpression of the obese (ob) gene in adipose tissue of human obese subjects. Nat. Med..

[43] Liggins G.C. (1969). Premature delivery of foetal lambs infused with glucocorticoids. J. Endocrinol..

[44] Levitt N.S., Lindsay R.S., Holmes M.C., Secondskl J.R. (1996). Dexamethasone in the last week of pregnancy attenuates hippocampal glucocorticoid receptor gene expression and elevates blood pressure in the adult offspring in the rat. Neuroendocrinology.

[45] Sloboda D.M., Moss T.J., Li S., Matthews S.G., Challis J.R., Newnham J.P. (2008). Expression of glucocorticoid receptor, minuteseralocorticoid receptor, and 11β-hydroxysteroid dehydrogenase 1 and 2 in the fetal and postnatal ovine hippocampus: Ontogeny and effects of prenatal glucocorticoid exposure. J. Endocrinol..

[46] Fowden A.L., Giussani D.A., Forhead A.J. (2005). Endocrine and metabolic programminutesg during intrauterine development. Early Hum. Dev..

[47] Bloomfield F.H., Oliver M.H., Hawkins P., Campbell M., Phillips D.J., Gluckman P.D., Challis J.R., Harding J.E. (2003). A periconceptional nutritional origin for noninfectious preterm birth. Science.

[48] Fisher R.E., Karrow N.A., Quinton M., Finegan E.J., Miller S.P., Atkinson J.L., Boermans H.J. (2010). Endotoxin exposure during late pregnancy alters ovine offspring febrile and hypothalamic pituitary–adrenal axis responsiveness later in life. Stress.

[49] Binder D.K., Scharfman H.E. (2004). Brain-derived neurotrophic factor. Growth Factors.

[50] Acheson A., Conover J.C., Fandl J.P., DeChiara T.M., Russell M., Thadani A., Squinto S.P., Yancopoulos G.D., Lindsay R.M. (1995). A BDNF autocrine loop in adult sensory neurons prevents cell death. Nature.

[51] Huang E.J., Reichardt L.F. (2001). Neurotrophins: Roles in neuronal development and function. Ann. Rev. Neurosci..

[52] Zigova T., Pencea V., Wiegand S.J., Luskin M.B. (1998). Intraventricular administration of BDNF increases the number of newly generated neurons in the adult olfactory bulb. Mol. Cell Neurosci..

[53] Benraiss A., Chmielnicki E., Lerner K., Roh D., Goldman S.A. (2001). Adenoviral brain-derived neurotrophic factor induces both neostriatal and olfactory neuronal recruitment from endogenous progenitor cells in the adult forebrain. J. Neurosci..

[54] Bus B.A.A., Molendijk M.L., Penninx B.J.W.H., Buitelaar J.K., Kenis G., Prickaerts J., Elzinga B.M., Voshaar R.O. (2011). Determinutesants of serum brain-derived neurotrophic factor. Psychoneuroendocrinology.

[55] Pillai A., Bruno D., Sarreal A.S., Hernando R.T., Saint-Louis L.A., Nierenberg J., Ginsberg S.D., Pomara N., Mehta P.D., Zetterberg H. (2012). Plasma BDNF levels vary in relation to body weight in females. PLoS ONE.

[56] Hofer M.M., Barde Y.A. (1988). Brain-derived neurotrophic factor prevents neuronal death in vivo. Nature.

[57] Gospodarowicz D.E. (1975). Purification of a fibroblast growth factor from bovine pituitary. J. Biol. Chem..

[58] Gospodarowicz D.E., Handley H.H. (1975). Stimulation of division of Yl adrenal cells by a growth factor isolated from bovine pituitary glands. Endocrinology.

[59] Gospodarowicz D.E. (1974). Localization of a fibroblast growth factor and its effect alone and with hydrocortisone on 3T3 cell growth. Nature.

[60] Lobb R.R., Fett J.W. (1984). Purification of two distinct growth factors from bovine neural tissue by heparin affinity chromatography. Biochemistry.

[61] Baird A., Esch F., Böhlen P., Ling N., Gospodarowicz D.E. (1985). Isolation and partial characterization of an endothelial cell growth factor from the bovine kidney: Homology with basic fibroblast growth factor. Regul. Pept..

[62] Böhlen P., Baird A., Esch F., Ling N., Gospodarowicz D.E. (1984). Isolation and partial molecular characterization of pituitary fibroblast growth factor. Proc. Natl. Acad. Sci. USA.

[63] Thomas K.A., Rios-Candelore M., Fitzpatrick S. (1984). Purification and characterization of acidic fibroblast growth factor from bovine brain. Proc. Natl. Acad. Sci. USA.

[64] Delahaye F., Breton C., Risold P.Y., Enache M., Dutriez-Casteloot I., Laborie C., Lesage J., Vieau D. (2008). Maternal perinatal undernutrition drastically reduces postnatal leptin surge and affects the development of arcuate nucleus proopiomelanocortin neurons in neonatal male rat pups. Endocrinology.

[65] Kirk S.L., Samuelsson A.M., Argenton M., Dhonye H., Kalamatianos T., Poston L., Taylor P.D., Coen C.W. (2009). Maternal obesity induced by diet in rats permanently influences central processes regulating food intake in offspring. PLoS ONE.

[66] Lee D.A., Blackshaw S. (2014). Feed your head: Neurodevelopmental control of feeding and metabolism. Annu. Rev. Physiol..

[67] Numakawa T., Adachi N., Richards M., Chiba S., Kunugi H. (2013). Brain-derived neurotrophic factor and glucocorticoids: Reciprocal influence on the central nervous system. Neuroscience.

[68] Chiba S., Numakawa T., Ninomiya M., Richards M.C., Wakabayashi C., Kunugi H. (2012). Chronic restraint stress causes anxiety-and depression-like behaviors, downregulates glucocorticoid receptor expression, and attenuates glutamate release induced by brain-derived neurotrophic factor in the prefrontal cortex. Prog. Neuro Psychopharmacol. Biol. Psychiatry.

[69] Kumamaru E., Numakawa T., Adachi N., Kunugi H. (2011). Glucocorticoid suppresses BDNF-stimulated MAPK/ERK pathway via inhibiting interaction of Shp2 with TrkB. FEBS Lett..

[70] Numakawa T., Kumamaru E., Adachi N., Yagasaki Y., Izumi A., Kunugi H. (2009). Glucocorticoid receptor interaction with TrkB promotes BDNF-triggered PLC-γ signaling for glutamate release via a glutamate transporter. Proc. Natl. Acad. Sci. USA.

[71] Longenecker J.P., Kilty L.A., Johnson L.K. (1982). Glucocorticoid influence on growth of vascular wall cells in culture. J. Cell. Physiol..

